# A Practical Tool for Risk Management in Clinical Laboratories

**DOI:** 10.7759/cureus.32774

**Published:** 2022-12-21

**Authors:** Jayagandan Jayamani, Chandrashekar C Janardan, Sadai V Appan, Kumaresan Kathamuthu, Manal Eldein Ahmed

**Affiliations:** 1 Laboratory, New Mowasat Hospital, Al-Salmiya, KWT; 2 Orthopaedics, Hywel Dda University Health Board, Wales, GBR

**Keywords:** failure modes and effects analysis, risk surveillance, risk mitigation, risk identification, risk assessment, laboratory risk management

## Abstract

Risk management constitutes an essential component of the Quality Management System (QMS) of medical laboratories. The international medical laboratory standard for quality and competence, International Standards Organization (ISO) 15189, in its 2012 version, specified risk management for the first time. Since then, there has been much focus on this subject. We authors aimed to develop a practical tool for risk management in a clinical laboratory that contains five major cyclical steps: risk identification, quantification, prioritization, mitigation, and surveillance. The method for risk identification was based on a questionnaire that was formulated by evaluating five major components of laboratory processes, namely i) Specimen, ii) Test system, iii) Reagent, iv) Environment, and v) Testing. All risks that would be identified using the questionnaire can be quantified by calculating the risk priority number (RPN) using the tool, failure modes, and effects analysis (FMEA). Based on the calculated RPN, identified risks then shall be prioritized and mitigated. Based on our collective laboratory management experience, we authors also enlisted and scheduled a few process-specific quality assurances (QA) activities. The listed QA activities intend to monitor new risk emergence and re-emergence of those previously mitigated ones. We authors believe that templates of risk identification, risk quantification, and risk surveillance presented in this article will serve as ready references for supervisors of clinical laboratories.

## Introduction

The test results generated by clinical laboratories aid in both diagnosis of patients' medical conditions and their continuous treatment monitoring. Since laboratory test results form an integral part of medical decisions, it becomes an absolute necessity that results generated by the lab are highly reliable and accurate. In recent years the field of laboratory medicine has shown tremendous technological advancements and automation in all of its major processes, which include pre-examination (pre-analytical), examination (analytical), and post-examination (post-analytical) processes. Despite the automation of processes, some risks are persistent, and those, if not controlled adequately, could result in a wrong diagnosis, wrong treatment, and ultimately morbidity and mortality. So identification and mitigation of potential risks associated with laboratory processes shall always be given prime importance. Risk management in laboratories, like any industry, follows similar strategies that include creating a process map that gives a better understanding of process flow among staff. Followed by which potential sources of errors are identified and assessed for their impact based on severity, the likelihood of occurrence, and detectability, implementing controls and checks to prevent and detect error before it harms the patient. This technical report is to emphasize that patients can be harmed not only by the issuance of erratic results but also due to failure of certain processes like delayed result generation or delayed critical results communication, etc.

## Technical report

Risk management and internal standards

The International standards organization (ISO) 3100:2018 standard on principles and generic guidelines on managing risks faced by organizations define risk as 'the effect of uncertainty on objectives'. Though this definition can be interpreted in different ways, in simple terms, it is the probability of an unfortunate occurrence. The standard ISO 15189:2012, which specifies the requirement of quality and competence in medical laboratories states, ‘The laboratory shall evaluate the impact of work processes and potential failures on examination results as they affect patient safety and shall modify processes to reduce or eliminate the identified risks and document decisions and actions taken’. It is evident from the above statement that the emphasis is on patient safety rather than risks arising due to lab safety issues, i.e., biological or chemical hazards. However, ignoring these risks could also have serious consequences on testing personnel and the testing environment to an extent causing a temporary or permanent shutdown of laboratories. So as a good laboratory practice and irrespective of accreditation status, risk management assessing all laboratory processes should be carried out to ensure both patients and personnel safety. Risk management is a cyclic preventive action that constitutes risk identification, risk quantification, risk prioritization, risk mitigation, and surveillance through a set of recurrent quality assurance activities [1].

Risk identification

A typical risk management activity starts from the identification of potential risks in current processes. There are three major processes within a clinical laboratory, namely pre-examination (pre-analytical), examination (analytical), and post-analytical. A logical approach for risk identification would be to map the steps within existing processes and evaluate for associated risks. We, the authors, developed a practical tool through the evaluation of the five components, namely i) Specimen, ii) Test system, iii) Reagent, iv) Environment, v) Testing personnel, and have developed a comprehensive seventy-point risk identification questionnaire which is listed in Table 1.

**Table 1:** Risk Identification Questionnaire ^1^PTS: Pneumatic Tube System; LIS: ^2^Laboratory Information System; ^3^IQCP: Individualized Quality Control Plan

S. No.	Risk identification questionnaire based on Good Laboratory Practices		Compliant (or) Partial compliance (or) Non-Compliant	Details of Non-compliance	
Pre-examination					
Specimen					
1	Are the formats, both electronic and physical, of test request forms complete in all aspects incorporating designated columns and rows to capture relevant clinical information as per international standards?				
2	Are the tests request forms completed by the requesting physicians filled with all relevant clinical information legibly?				
3	Whether the lab have a procedure to accept the clinical specimens that are collected in a location outside hospital premises, e.g., Homecare services or from other clinics or hospitals? (excluding timed specimen collections like 24 hours Urine stool, etc.)				
4	Whether the directory of testing services (including 'send out' tests) readily available at the front office counters?				
5	Whether a queue management system is available at the front office and specimen collection area to reduce overcrowding and reduce waiting time?				
6	Whether an adequate number of chairs are available in the waiting area?				
7	Whether adequate measures are taken for giving priority to pediatric patients and special needs patients?				
8	Whether vein viewers are available for quick identification of veins in obese and pediatric patients?				
9	Whether the policy for positive patient identification clearly understood and compliance monitored?				
10	Whether all front office and phlebotomy staff are aware of standard precautions, infection prevention and control measures, and emergency codes?				
11	Are all staff aware of the specimen types, order of draw of tubes with anti-coagulants and preservatives, and transport requirements?				
12	Are all types of specimen containers procured and stored in adequate quantity for providing uninterrupted service?				
13	Is there a mechanism to monitor the expiry of all consumables in all specimen collection areas?				
14	Whether specimen packaging and transport are carried out adhering to established infection control procedures?				
15	Whether all staff is aware of the spill management protocol?				
16	Whether primary specimens received are processed (e.g., centrifuged) and secondary samples separated on time?				
17	Whether procedures like sample centrifugation, separation, and storage are carried out according to manufacturer recommendations?				
18	Whether adequate measures taken to reduce the delay in receipt and acknowledgment of those specimens sent through the Pneumatic tube system (PTS)^1^?				
19	Whether specimen rejection criteria are established and followed?				
20	Whether procedures for repeat sample collection are clearly defined for both out-patients and in-patients departments?				
21	Whether procedures related to collection, storage, packaging, and transport available for sending out/referring specimens?				
22	Whether adequate preventive measures are taken to tackle situations like lost sample shipments during transit to the referral lab?				
Examination					
Equipment					
23	Whether all equipment involved directly or indirectly involved in testing is accepted after proper validation and result comparison studies?				
24	Whether all laboratory equipment receives scheduled (Daily, Weekly, Monthly, and quarterly) maintenance on time?				
25	Whether the laboratory has maintenance contracts for all the critical equipment?				
26	Whether all testing equipment have an inherent technology to alert the users about failures encountered during barcode reading, aspirating, dispensing, calibrating, quality control analysis, and any electrical or electronic, or mechanical failures?				
27	Whether the laboratory have a procedure to verify equipment and applicable assay calibrations?				
28	Whether the laboratory have a quality control plan meeting regulatory bodies’ recommendations?				
29	Whether the laboratory uses sufficient levels of quality control materials to cover the reportable ranges?				
30	Whether the laboratory participates in proficiency testing programs covering the scope of testing services?				
31	Whether the laboratory conducts a proper root-cause analysis and documents corrective and preventive actions for internal quality control and proficiency testing failures?				
32	Whether the laboratory have an information management system to relay electronic test requests to testing equipment and receive test results?				
33	Whether the laboratory verifies periodically the data interfaced to and from Laboratory Information System (LIS)^2^?				
34	Whether LIS access is protected and has an audit trail of all events?				
35	Whether backup plans are in place for handling unexpected power or internet failures or server breakdowns due to whatsoever reason?				
Reagents					
36	Whether the laboratory store sufficient stock of reagents for uninterrupted service?				
37	Whether reagents received by the laboratory are checked for continuity of cold chain, longer or sufficient expiry?				
38	Whether the laboratory have a mechanism to identify the reagents of different lots and shipments?				
39	Whether the laboratory verifies each new lot of reagents and calibrators using appropriate quality control procedures?				
40	Whether the laboratory have a procedure to verify and validate all newly installed equipment, new methods, newly introduced tests, etc.?				
41	Whether the laboratory have adequate and appropriate storage facilities like refrigerators, freezers, and designated store rooms for lab reagents and consumables?				
42	Whether laboratory store highly toxic and dangerous chemicals in a designated protected area with appropriate safety precautions?				
Environment					
43	Whether the laboratory have adequate space to conduct safely the testing activities?				
44	Whether the laboratory is strategically located to cater faster service to emergency rooms, intensive care units, and operation theatres?				
45	Whether the laboratory is well-lit and adequately ventilated for conducting all phases of testing services?				
46	Whether all instruments are placed fulfilling the manufacturer's installation requirements?				
47	Whether the laboratory have a sufficient number of water plants for an uninterrupted supply of deionized water for the wet chemistry lab?				
48	Whether the laboratory is equipped with surge protectors, the appropriate type of fire extinguishers, smoke detectors, and sprinklers in adequate numbers?				
49	Whether the laboratory solid wastes cleared off from testing areas at appropriate intervals?				
50	Whether the laboratory liquid wastes disposed of in compliance with local regulations?				
Testing Personnel					
51	Does all technical staff, including phlebotomists, technologists, and lab physicians, have suitable qualifications and experience and are licensed by the local regulatory authority to practice their field of expertise?				
52	Do all personnel receive the necessary training relevant to their area of work?				
53	Are all new personnel subjected to a systematic training and orientation program before assigning individual responsibilities?				
54	Are all technical personnel aware of the Individualized Quality Control Plan (IQCP)^3^ of their working area?				
55	Do all Technologists receive training on basic internal quality control troubleshooting procedures and documentation?				
56	Are all personnel trained about safe laboratory practices and notification of adverse occurrences?				
57	Do all personnel receive job training relevant to their nature of work to keep abreast with current advancements in testing services?				
58	Do all personnel has defined job responsibilities?				
59	Are all personnel subjected to periodic competency assessments?				
60	Whether the laboratory have an annual training plan based on the identified training needs?				
Post-examination					
61	Whether the laboratory have a documented procedure for auto-verification and two-level verifications for the release of other non-auto-verified reports?				
62	Whether the laboratory includes all relevant information in its reports essential for clinical interpretation?				
63	Whether the laboratory have a procedure to identify and notify the critical results of the clinical team?				
64	Whether the laboratory have a mechanism to communicate or indicate additional comments along with lab results?				
65	Whether the laboratory have a protective mechanism to prevent tampering with released results?				
66	Whether the laboratory have a procedure for the amendment or recall of reports released to patients?				
67	Whether the laboratory adopt a clear policy for releasing the results of referred tests?				
68	Whether the laboratory adopt a clear policy for issuing hardcopy reports to caretakers instead of patients?				
69	Whether the laboratory defined its retention period for specimens and records?				
70	Whether the laboratory monitors test turnaround times periodically?				

Risk quantification

Risk quantification is a process of evaluating identified risks, which is the sentinel step in making the right decisions as to what needs to be done to remove or reduce the chance of its occurrence. Identified risks shall be quantified using the FMEA tool, which is based on the calculation of the RPN. RPN is a multiplication product of scores assigned for severity (S), likelihood of occurrence (O), and likelihood of detection (D), and Table 2 contains the details of the scoring assignment system [2-3].

**Table 2:** Scores for Risk Quantification ^1^Severity (S): If a risk leads to error, how severe it could harm the patient? Scores are assigned between 1 and 10, which means that risks with a score of 1 are very unlikely to harm the patient, and those assigned a score of 10 are very likely to cause severe harm (morbidity or mortality). ^2^Likelihood of occurrence (O): How likely is the identified risk that could lead to error? Scores are assigned between 1 and 10, which means that for risks with a score of 1, errors are least likely or rare to occur, and for those assigned a score of 10 errors can occur very frequently. ^3^Likelihood of Detection (D): How likely are the errors will be detected? Scores are assigned between 1 and 10, which means errors with a score of 1, are easily detectable/obvious, and those assigned a score of 10 are completely not-detectable [3].

Severity (S)^1^		
Effect	Rank	Criteria
No	1	No effect
Very Slight	2	Customer not annoyed
Slight	3	Customer slightly annoyed
Minor	4	Customer experiences minor nuisance
Moderate	5	Customer experiences some dissatisfaction
Significant	6	Customer experiences discomfort
Major	7	Customer dissatisfied
Extreme	8	Customer very dissatisfied
Serious	9	Morbidity
Hazardous	10	Mortality
Likelihood of Occurrence (O)^2^		
Probability of Failure	Rank	Criteria
Very Low	1	Failure eliminated preventative control already in place
Low	2	Failure never occurred with current process
Low	3	Once in 5 to 10 years
Moderate	4	Annually
Moderate	5	Biannually
Moderate	6	Quarterly
High	7	Monthly
High	8	Weekly
High	9	Daily
Very High	10	Multiple times within a day
Likelihood of Detection (D)^3^		
Certainty	Rank	Criteria
Almost certain	1	Evident
Very High	2	Familiar alert system in place
High	3	Unfamiliar alert system in place
Moderately High	4	Recurrently failing alert system
Moderate	5	Alert system entirely dependent on subjective interpretation
Low	6	Low chance of detectability
Very Low	7	Very low chance of detectability
Remote	8	Rarely existing process can identify
Very remote	9	Very rarely existing process can identify
Almost uncertain	10	No mechanism to identify the defect

Risk prioritization

In simple terms, it is the process of determining the sequence in which the identified risk will be acted upon. It may appear easier at first glance that this task of assigning priority could easily be achieved by looking at the RPNs. It is a tough task if clusters of risks will be identified and all of them have the same RPNs. In general, most rational approaches are based on answering a few questions, such as How critical it is in compromising patient safety? How imminent is the error? What impact it could cause immediately and in the future on the reputation of the Laboratory? What are the expected financial losses to the Laboratory? Whether the risk has the potential to recur within the period of non-attention when it has not received priority? [4]. All the risks identified shall be listed using the template, as shown in Table 3.

**Table 3:** Combined Risk quantification and re-quantification table ^a ^S: Severity; ^b ^O: Likelihood of Occurrence; ^c ^D: Likelihood of Detection; ^d ^RPN: Risk Priority Number

Risk Quantification							Mitigation			Re-quantification				
S. No.	Risks identified	Potential errors	S^a^ (1 to 10)	O^b^ (1 to 10)	D^c^ (1 to 10)	RPN^d^ (S*O*D)	Corrective actions required when RPN ≥100	Recommended corrective actions	Corrective action completion date	S^a^ (1 to 10)	O^b^ (1 to 10)	D^d^ (1 to 10)	RPN^d^ (S*O*D)	Risk mitigated (Yes / No)

Risk mitigation

This step of risk management involves the planning and development of methods and options that are focused on reducing the risks and errors that might occur as a consequence of a risk. There are three important strategies of risk mitigation that are specifically applicable to healthcare settings, namely 1) Avoid: This type of strategy is usually adopted when identified risks have to be completely eliminated as it may have direct effects on the reputation of the organization. For example, suspending routinely requested tests due to the non-availability of reagents or consumables. For example, sudden suspension of cardiac markers or any similar critical testing can have a major negative impact on a patient's clinical management, and labs are always expected to have contingency plans in place for such testing services. 2) Control: This strategy applies to those risks that are repetitive and can never be completely eliminated as they are inherent to an existing system of operation. A classical example in a laboratory for this strategy is analyzing quality control materials before analyzing the patient samples. It is the responsibility of the laboratory to decide upon the frequency of such analysis to keep control of the risk of generating and releasing erratic test results. 3) Watch/Monitor: This strategy is watching and identifying any changes that can affect the impact of the risk. For example, during the COVID-19 pandemic, many healthcare organizations took a risky decision to invest in setting up a molecular diagnostic laboratory, the reason why it is risky is that governments had decided to price cap COVID-19 testing and poorly planned resource allocation could have led to huge financial losses. Another similar example is at the start of the pandemic, there were many rapid card-based test methods with limited or unknown sensitivity, and specificity was used widely. Hospitals that could not afford the state of the art of molecular method opted to use these rapid tests. In such a scenario, watchful surveillance of predictive values of such card-based tests becomes essential [5].

Surveillance

Risk surveillance is a thorough set of quality assurance activities carried out in a scheduled manner, either through brainstorming sessions with the risk management team members or by adopting recommendations of regulatory bodies or published guidelines. The quality assurance activities, as listed in Table 4, could be as small as logging housekeeping of laboratory to hospital server backup activities. Surveillance helps to continuously evaluate the effectiveness of completed risk management activity.

**Table 4:** Scheduled Quality Assurance activities P: Indicates to perform as per indicated schedule.

S. No.	Quality assurance activity	Minimum Frequency				Assessment of activity (Compliant with established limits) Yes / No	Corrective actions (if applicable)	Remarks
		Daily	Monthly		Annually			
Pre-examination								
1	Front office waiting time surveillance		P					
2	Phlebotomy waiting time surveillance		P		P			
3	Logging sample details rejected for analysis	P	P		P			
4	Review of requests pending specimen collection	P						
5	Keep track of samples to be sent out to the referral lab	P						
6	Competency assessment for phlebotomists				P			
7	Customer satisfaction index monitoring on reception and phlebotomy services		P					
Examination								
8	Equipment downtime monitoring			P	P			
9	Temperature monitoring of all refrigerators, freezers, and general storage area and additional humidity monitoring for testing locations	Thrice a day						
10	Review of internal quality control data	P						
11	Review of Levey Jenning Charts			P				
12	Sigma metric calculations for applicable tests				P			
13	Review of proficiency testing performance	P		P	P			
14	New lot (reagents, calibrator, and calibration verification) and disposal of expired reagents & consumables	As the need arises						
15	New method verifications and validations	As the need arises						
16	Comparison of point-of-care testing methods with lab method for applicable tests	Twice weekly						
17	Proficiency testing for point-of-care tests	Quarterly						
18	Point of care testing, monitoring compliance on established policy and procedure			P	P			
19	Competency assessment for technical staff				P			
20	Continual professional development for technical staff			P				
Post-examination								
21	Sample archival and disposal as per policy	P						
22	Critical alert logging and surveillance	P		P	P			
23	Review of samples pending analysis after their receipt in the lab	P						
24	Surveillance of the analytical turnaround times of out-patient samples			P	P			
25	Surveillance of the analytical turnaround time of emergency room samples			P	P			
26	Review of results of referred samples	P						
27	Surveillance of turnaround times of samples sent to a referred lab			P				
28	Surveillance of report amendments			P				
Management requirements								
29	Document revision in line with updates to standards or changes in current policies				P			
30	Surveillance and review of the performance of the established quality indicator			P	P			
31	Laboratory review meeting			P				
32	Point of care testing committee meeting	Quarterly & as the need arises						
33	Liaison with technical heads of referral lab to review service agreements				P			
34	Customer feedback survey on laboratory services				P			
35	Vendor evaluation				P			

## Discussion

The procedure typically starts by forming a risk management committee constituting key personnel of the Laboratory and a few Adhoc members at the discretion of the main members. The committee shall perform a process flow analysis of all laboratory technical processes (pre-examination, examination, and post-examination). Evaluate the five major components of the testing process, namely Specimen, Test system, Reagent, Testing Environment, and Testing personnel, to identify potential sources of errors. In this regard the committee shall meet regularly for brainstorming sessions to review the laboratory’s established policies, standard operating procedures, and work instructions; feedback about the front office and phlebotomy services; annual feedback from internal customers (hospital physician and nurses); performance of established quality indicators; equipment records, including major and minor breakdown logs; records of the callback of supplied reagents, calibrators or quality control lots; performance in proficiency testing programs; vendor evaluation records; adverse occurrence/incidents reports; personnel employee training records and training needs assessments.

**Figure 1:**
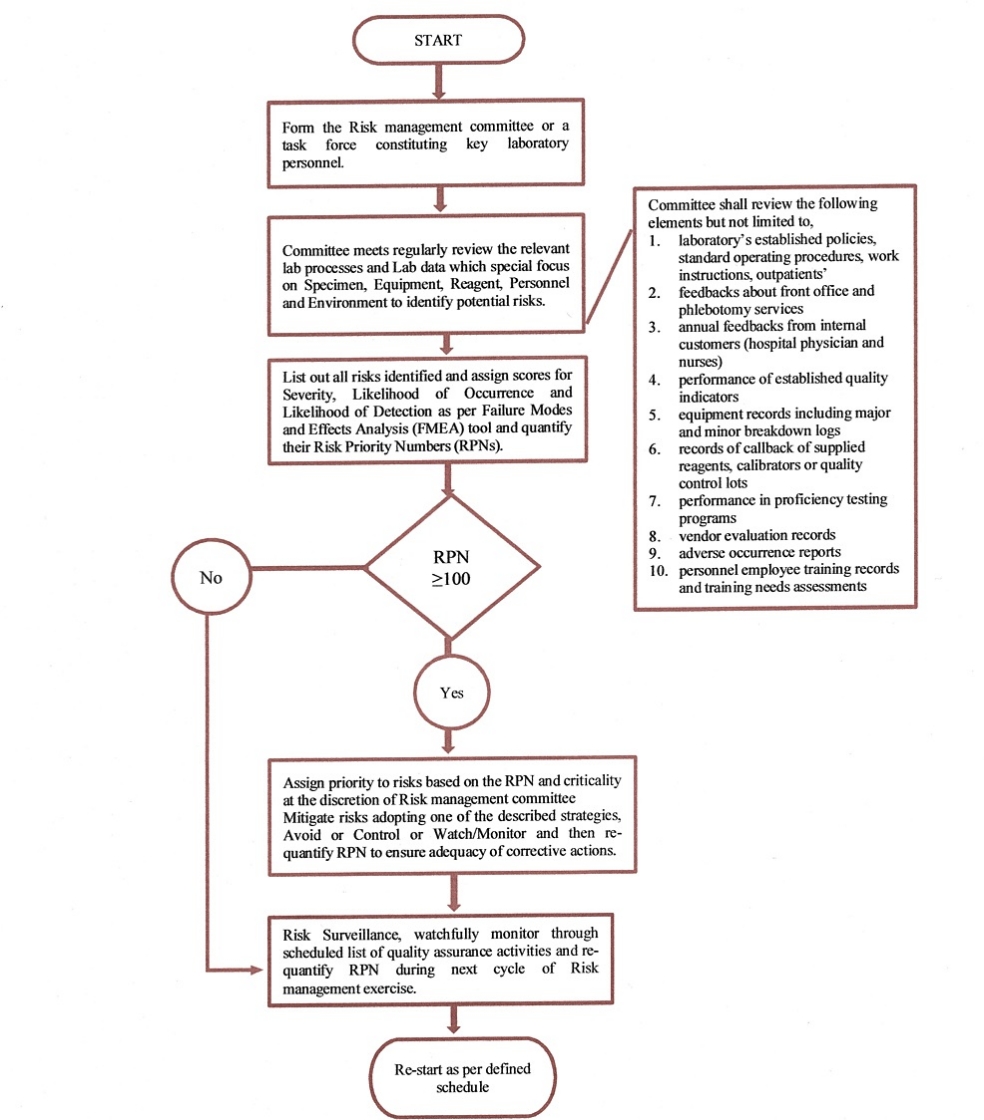
Risk Management Process Flow

The current paper presents a seventy-point questionnaire listed in Table 1 that shall serve as a readymade template for laboratorians to complete the risk identification step. This article also presents Table 2, which aids in score assignments for risks identified, and Table 3, which gives a template for initial RPN calculation and recalculation following the implementation of corrective actions. Table 4 presents a list of scheduled quality assurance activities that helps not only in controlling the re-emergence of already mitigated risks but also in the emergence of any potential risks. Figure 1 demonstrates a typical flow of the described risk management procedure.

There are pieces of literature quoting RPN targets between 300 to 1000, but in a healthcare setting, any risk with RPN greater or equal to 100 has the potential to cause an error and should be eliminated [6]. On the other hand, it is also quite possible that a few of identified risks may have RPN less than 100, but the severity score assigned might be 9 or 10. Such risks should not be ignored based on low RPN only, and control measures should be devised and implemented to eliminate them from the process. 

Limitations

Though FMEA-based risk management is considered highly practical and prevalent, there are certain subjective components to it. For example, score assignments for the severity of identified risks are subjective and might directly affect the RPN calculation [7]. There are chances for both under and overestimation of RPN. From the flow of risk management described in Figure 1, it is evident that RPN is the deciding factor of risk mitigation and should be accurate. Table 2, containing the scores and the criteria, to a greater extent, helps to avoid the subjective component in score assignments.

## Conclusions

Risk management, though it originates from manufacturing industries, is not a newer concept in healthcare settings. As we are in the era of evidence-based medical practice, there has been a lot of focus on this topic. There are plenty of published pieces of literature on risk management in health information protection, healthcare process management, in-vitro diagnostics production, pharmaceutical production, drug dispensing, and so on. In developed nations, risk management forms an essential component of their patient safety program. Risk management in clinical laboratories is an essential quality improvement activity that must evaluate all processes involved in testing. It is a repetitive preventive action that needs to be carried out by laboratories at least annually. The risk identification questionnaire demonstrated in this article is easy to understand and implement and can be assigned to any supervisory staff for completion without needing additional training. Similarly, the Quality Assurance (QA) activities enlisted are quite comprehensive and include both technical elements and general management elements. The schedules for QA activities are majorly classified into daily, monthly, and annual basis are clearly indicated in the text. These identified QA activities serve as tools for both risk surveillance and capturing quality indicator data of laboratory processes.

As a continuous improvement initiative, these QA activities shall be assessed, and appropriate actions shall be planned, implemented, and documented. We authors, through this report, have tried to provide a sustainable solution to laboratory supervisors for carrying out risk management exercises. 
